# Small droplet emission in exhaled breath during different breathing manoeuvres: Implications for clinical lung function testing during COVID‐19

**DOI:** 10.1111/all.14596

**Published:** 2020-10-06

**Authors:** Neil J. Greening, Per Larsson, Evert Ljungström, Salman Siddiqui, Anna‐Carin Olin

**Affiliations:** ^1^ Department of Respiratory Sciences University of Leicester Leicester UK; ^2^ Institute for Lung Health NIHR Biomedical Research Centre Glenfield Hospital Leicester UK; ^3^ Unit of Occupational and Environmental Medicine Department of Public Health and Community Medicine Institute of Medicine Sahlgrenska Academy University of Gothenburg Gothenburg Sweden; ^4^ Atmospheric science Department of Chemistry and Molecular Biology University of Gothenburg Gothenburg Sweden

To the Editor,

Transmission of severe acute respiratory syndrome coronavirus 2 (SARS‐CoV‐2) within the healthcare setting is a major concern for infection prevention. In particular concern has been noted for small droplets (typically ≤ 5 microns), which have been shown to contain virus,^1^ as well as remaining airborne for longer, potentially increased spread.^2^ Modelling has shown particular spread of particles once exhaled during movement and exercise,^3^ but increases in exhaled particle mass are less well established, and likely to vary depending on breathing pattern.

Pulmonary function testing (PFTs) is essential to respiratory medicine, and COVID‐19 has had considerable implications.^4^ International guidance highlights PFTs as an aerosol generating procedure, but to date the varying response of different breathing manoeuvres have not been considered (see supplemental Figure [Link], [Link]). The use of in‐line filters is standard of care in many lung function labs, reducing the risk of viral and bacterial contamination.

Quantification of respiratory tract lining fluid (RTLF) in exhaled breath is possible and has been shown to represent both particles originating in the small airways, as a result of liquid bridge rupture following airway closure, and particles within the central airways, due to shear forces and airway wall flutter.^5^

We aimed to determine the mass of small droplets exhaled at varying flow rates and during different respiratory manoeuvres in healthy participants, simulating different lung function tests commonly used in practice, using tidal breathing and cough as anticipated minimal and maximum manoeuvres.

We quantified particle formation during different breathing manoeuvres using Particles in Exhaled Air (PExA, Sweden).^6^ Manoeuvres were performed using particle free HEPA filtered inspiratory air, but no particle filter between exhalation and sampling. We reanalysed data from healthy volunteers (mean (SD) age 46(17) years, body mass index 23.9 (2.9) kg/m^2^, FEV_1_ 101.8(11.0) % predicted) using both previously published and unpublished data.^7^, ^8^ Studies were approved by the Ethical Review Board at the University of Gothenburg.

Breath manoeuvres included; (A) tidal breathing (TV), (B) forced expiratory volume (FEV), (C) slow vital capacity following inspiration from functional residual capacity (sVC‐FRC), (D) sVC following inspiration from residual capacity (sVC‐RV) and (E) cough at total lung capacity (see supplemental figure). Data for the different breathing manoeuvres were obtained from a total of 33 healthy volunteers and were included in this analysis (22 for tidal volumes, 11 for all lung function tests). Maximal flow rate and particle mass were available from 102 participants.

There was minimal increase in particle mass during tidal breathing (A) compared with background noise (median mass per litre of breath 0.09ng/l [IQR 0.09]). FEV (C) resulted in a higher particle mass production than tidal breathing or sVC following FRC (B) (+150%, 95%CI 10‐470, *P* = .03) vs sVC‐FRC, but less than other manoeuvres (Figure 1).

**Figure 1:**
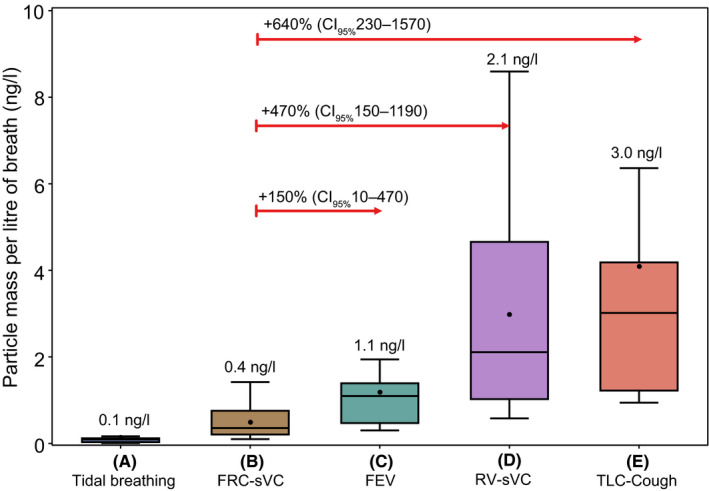
Mass of exhaled particles per breath using PExA following different breathing manoeuvres. Median values are shown above each plot. Box and whisker plots are median (line), mean (dot) with interquartile range (box) and range (whiskers)

sVC particle mass varied depending on prior inspiratory manoeuvre, with minimal difference to tidal breathing when performed from FRC (B). A significant increase in particle mass was seen with sVC following inspiration from RV (D) (+470%, 95%CI 150‐1190, *P* < .01), compared with sVC‐FRC, likely secondary to small airway RTLF liquid bridge rupture following airway closure and reopening.

Coughing (E) resulted in the highest mass of exhaled particles compared with all other manoeuvres, with a 640% (95%CI 230‐1570, *P* < .01) increase compared to sVC‐FRC (C) (Figure 1).

We also observed no difference in mass of small particles with change in peak expiratory flow rate (coefficient −1,16, *P* = .221) (Figure 2), when analysing data from a larger data set (n = 107) where expiratory flow have been measured. Of note, the expiratory flow was lower (20‐200 l/m) than in forced exhalations (typically 250‐600 L/m).

**Figure 2:**
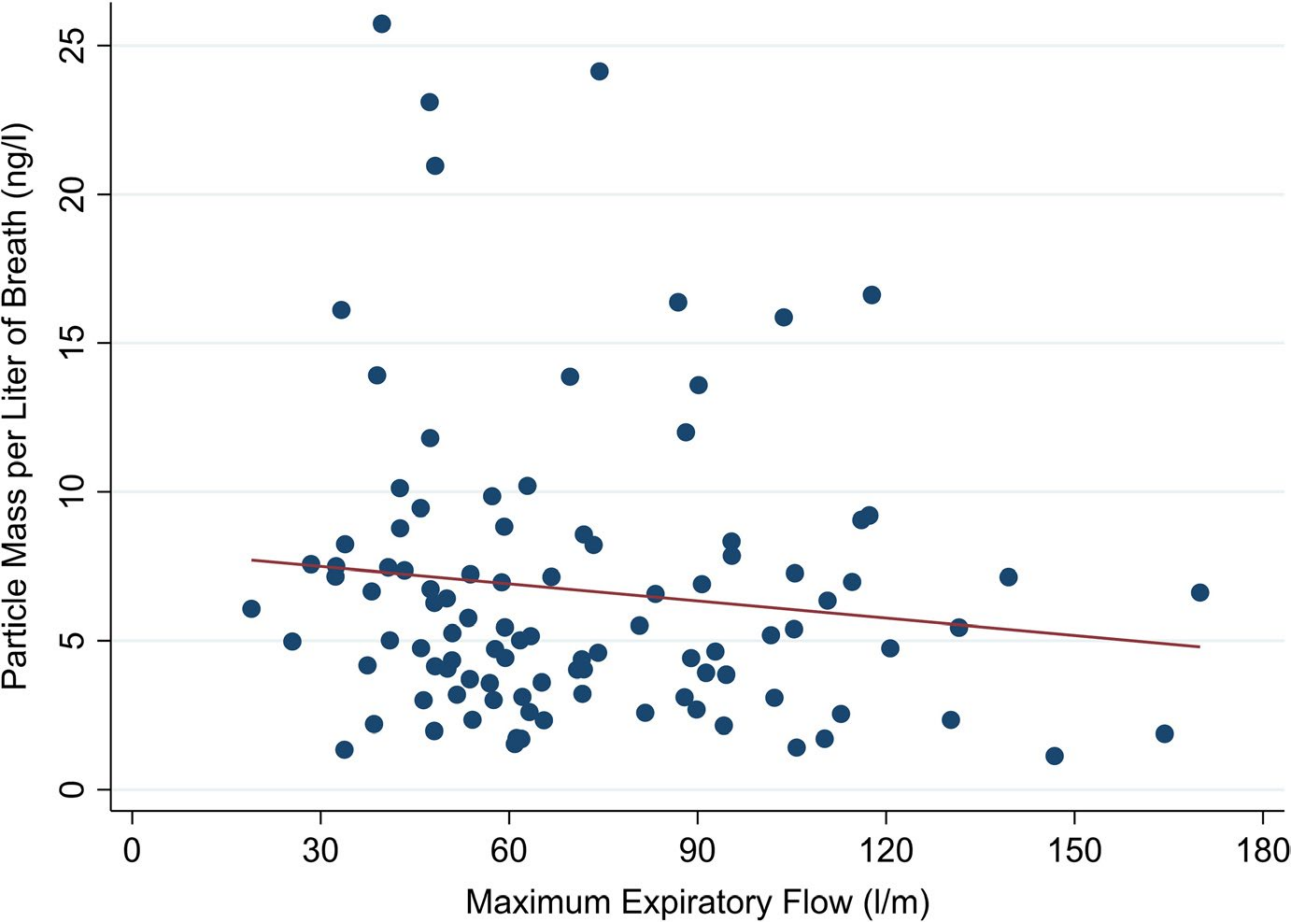
Scatterplot of mass of exhaled particles per breath using PExA in relation to maximum exhalation flow rate. No significant relationship with flow rate was seen (Linear regression line shown in red)

We demonstrate that exhaled small particle mass varies with different breathing manoeuvres, with minimal production during tidal breathing. Mass of exhaled particles did not increase with increasing exhalation flow, suggesting that during co‐ordinated breathing manoeuvres small droplet mass is related to total ventilation volume, and formed predominantly from airway opening.

Coughing is associated significant increase in particles due to involuntary and uncoordinated airway spasm, resulting in greater RTLF film rupture, as well as upper airways shearing. This is likely to confer significant risk during PFTs, and procedures such as sputum induction or physiotherapy, for example using hypertonic saline.

These data have implications for different breathing manoeuvres performed during PFTs. Lung function remains essential for both the diagnosis (eg FEV_1_ for COPD) and therapeutic response of medication (eg VC in IPF, FEV_1_ in cystic fibrosis) in respiratory medicine. Spirometry, in the absence of coughing, is likely not to present a considerably higher risk above background risk, in the absence of an airway closure manoeuvre prior to the VC test.

For measures in confirmed airways disease, such as asthma and COPD, these data suggest that fewer droplets would be produced, potentially reducing risk, if a FEV manoeuvre was stopped once achieved and before exhalation to RV. This would not be in keeping with current lung function standards, but would result in a “usable curve” and an acceptable measure that could be used clinically.

These results do suggest that the inspiratory manoeuvre before a VC is important to reduce small particle production. This may be particularly relevant for healthcare staff supporting patients performing lung clearance and airways breathing techniques during acute illness, with the increase in particle release during deep inhalation and holding manoeuvres.

The risk of viral transmission from the small particles is unclear as the precise relationship between particle size and viral RNA content are uncertain. However, the risk of lung deposition and sedimentation will be higher with smaller particles^2^ and are associated with up to 65% of viral load.^9^ Most lung function laboratories will use in‐line filters, to prevent contamination, based on ATS/ERS standards, meaning air droplets are effectively filtered in these cases. Our data strengthen their mandated use, but also highlights potential small droplet exhalation in the breath following spirometry (as from RV), which are usually into the atmosphere.

The manoeuvres in this study do not meet current ERS/ATS spirometry standards. However, with many lung function labs unable to perform tests at all during the COVID pandemic, further data and solutions are required. Usable, but not acceptable, manoeuvres that potentially reduce infection risk may be required when there is risk of infection. We would hope this work may help inform updated guidelines for ERS/ATS. Furthermore, our data suggest that first fewbreaths immediately following the measurement should also exhaled into a filter before letting go of the mouthpiece, ensuring particles continue to be exhaled into filters.

Limitations of the study include that results were obtained from an adult healthy population and may be different in those with lung conditions, in particular those with mucus hypersecretion. However, it is important to first understand particle formation in health. Previous data suggest that small droplet formation may be lower in patients with asthma,^6^ though the effects during PFTs are unknown. Also, the PExA system registers mostly small droplets from the small airways, and virus are likely to be present in both upper and lower respiratory droplets. We have previously demonstrated that droplets produced during coughing had very little surfactant content, suggesting upper airways origin.^7^ Manoeuvres also included a breath hold before exhalation for B,C and E which may result in lower flow rate and particle release. This could potentially as a technique to reduce particle release be used for nonflow rate manoeuvres (eg sVC), but this would need to be confirmed in further studies.

In summary, we show that small droplet emission varies for different breath manoeuvre performed during PFTs, with very low production in TV and sVC from FRC and low production during FEV. Consideration of performing PFTs in different clinical settings could account for these differences, with future focus of clinical risk also on room ventilation.

## CONFLICT OF INTEREST

PL, EL and AO are shareholders in PEXA® AB (www.PEXA.se). AO and EL has a patent associated with the PEXA® method (Patent number: wo2009045163). SS has received grants for PEXA research from the Chiesi Onulus foundation. No other conflict of interest is declared by the authors.

## Supporting information

Fig S1

Supplementary Material
