# Supplementary figures and images for: Small droplet emission in exhaled breath during different breathing manoeuvres: Implications for clinical lung function testing during COVID‐19
Source: Allergy. 2020 Oct 6;76(3):915–7. doi: 10.1111/all.14596 (PMC7537081; doi:10.1111/all.14596)

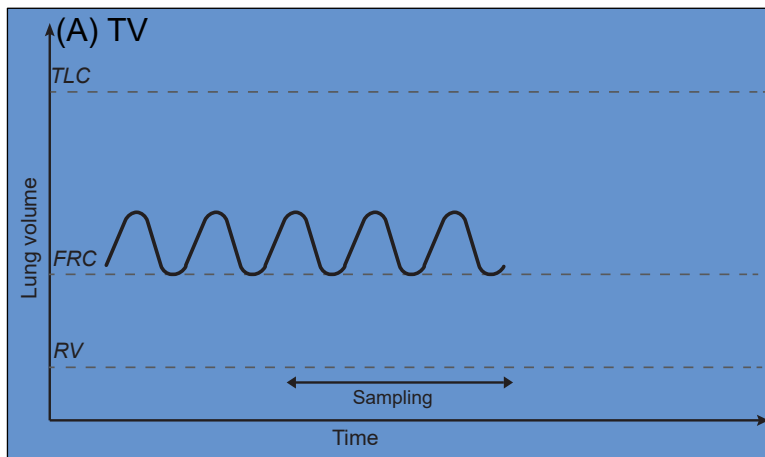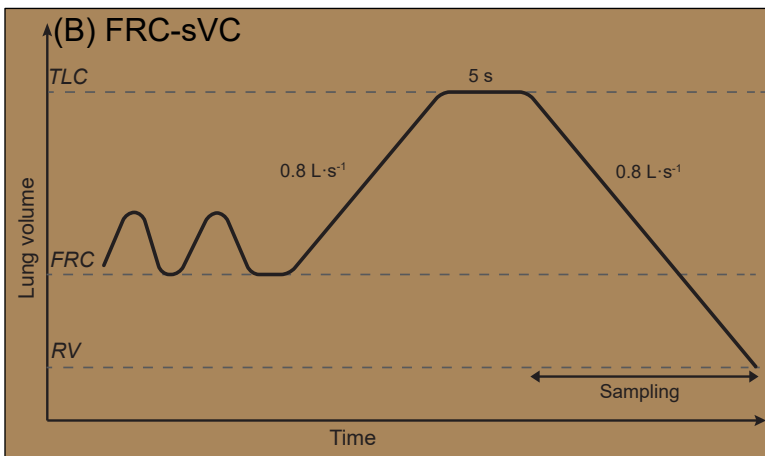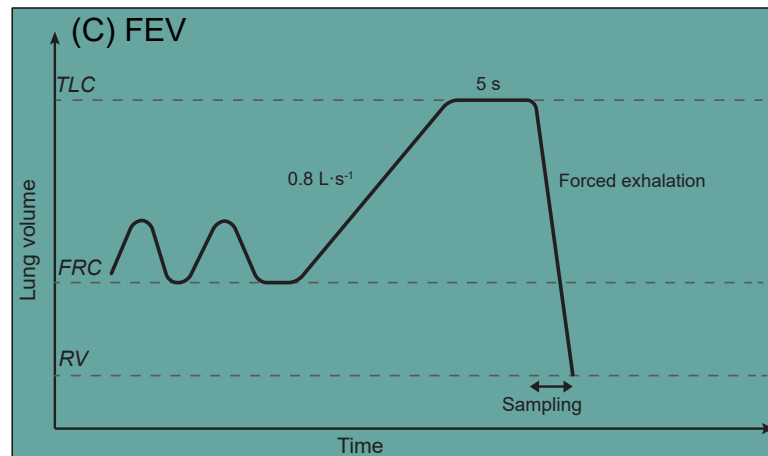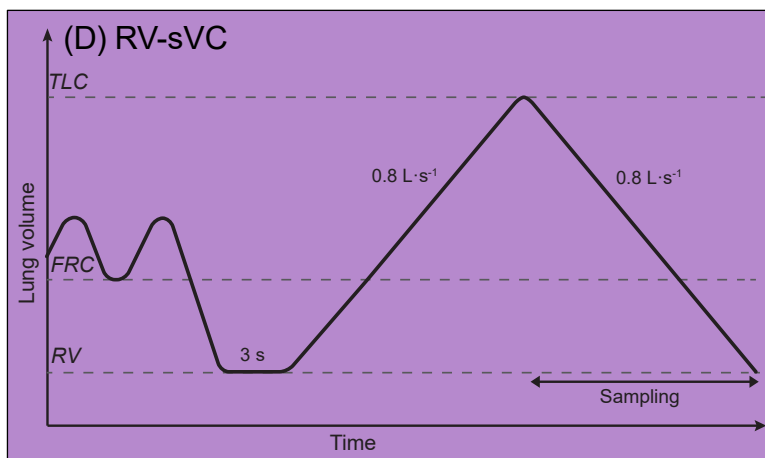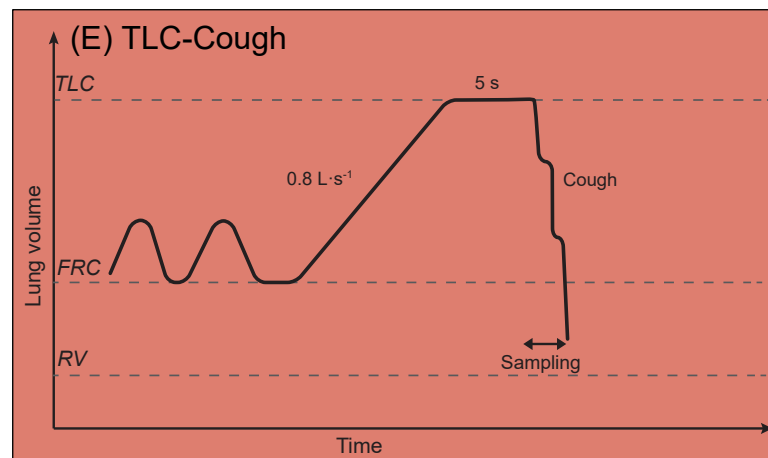

Supplement: Supplementary file 1 — Fig S1 [file ALL-76-915-s001.pdf]
