# Supplementary material for: Small droplet emission in exhaled breath during different breathing manoeuvres: Implications for clinical lung function testing during COVID‐19
Source: Allergy. 2020 Oct 6;76(3):915–7. doi: 10.1111/all.14596 (PMC7537081; doi:10.1111/all.14596)
Supplement: Supplementary file 2 — Supplementary Material [file ALL-76-915-s002.docx]

*Supplemental Figure: Different lung function manoeuvres tested in this paper, including sampling point and pre-sampling breathing. (A) Tidal Volume (B) slow Vital Capacity following inspiration from Functional Residual Capacity (C) Forced Expiratory Volume (D) slow Vital Capacity from Residual Volume (E) Cough from Total Lung Capacity*
